# ^Woa-wtconv-kanformer for long term time series forecasting^

**DOI:** 10.1371/journal.pone.0340805

**Published:** 2026-01-23

**Authors:** Meng Ling Ming, Qi Wei Min, Dong Yi Fan, Zheng Yu Ning

**Affiliations:** 1 School of information department, Hebei Youth Management Cadre Institute, ShiJiaZhuang, HeBei, China; 2 School of Artificial Intelligence, Jianghan University, WuHan, HuBei, China; Kafkas University: Kafkas Universitesi, TÜRKIYE

## Abstract

Multivariate time series analysis and prediction are of great significance in traffic management, weather forecasting and other practical applications. However, most of the existing research focuses on using the traditional transformer model as the framework to predict short series or predict with time domain features, and the effect of removing noise interference with irregular frequency is not good. At the same time, because the model based on the transformer framework needs to adjust too many hyperparameters, improper parameter design in practical use will lead to model performance degradation, so we propose the WOA-WTConv-KANformer model. The model is optimized based on the itransformer time series prediction model. Before embedding the time nodes of each series into the variable token and input into the encoder layer, the WTConv2d model is used to process the data with wavelet frequency to extract the frequency domain and time domain features. So that the model can solve the non-stationarity problem of time series data caused by the frequency domain problem. In order to realize the effective training of the model, we also use the whale optimization algorithm to make the model reasonably adjust the hyperparameters before formal training. At the same time, the KAN module is used as the linear layer instead of MLP during the training and use of the model, so that the model can improve the performance of different prediction lengths. The number of training parameters is also reduced. Through five public prediction datasets, it is shown that our model can achieve performance improvement on different prediction lengths, and the training efficiency is also improved, which proves the potential of the model in the field of real-world time series prediction.

## 1. Introduction

Time series data are widely used in numerous real-world scenarios for instance finance, medical care, and meteorology, and are ubiquitous in real-world applications that require effective analytical methods [1] to deal with data streams of multiple interrelated variables, also known as multivariate time series problems. Therefore, understanding and analyzing history-based multivariate time series, and processing them for forecasting, has sparked great interest in various practical applications. In recent years, the rapid advancement of deep learning has significantly transformed the field of time series analysis. These advancements have enabled significant breakthroughs across a wide range of applications, including predictive analytics [2–3], interpolation [4–5] and anomaly detection [6–7] and other related applications of temporal processing.

**Fig 1:**
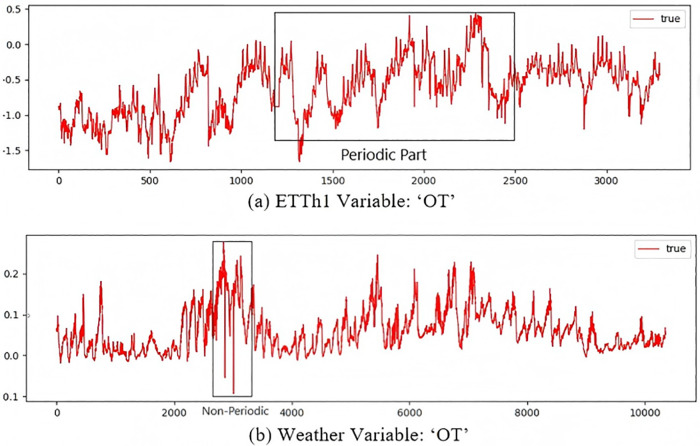
Real-world time series with distinct periodic pattern.

However, even with the good performance of the above methods, real-world time series show different periodic patterns. An important limitation of research in this area is that nonstationarity has changing statistical properties and time dependent patterns, it presents a significant challenge to traditional deep learning models [8–9]. As shown in Fig 1, the dynamics and complexity of real-world time series data often lead to models failing to effectively capture these changing patterns, and periodic time domain features usually exhibit more global dependencies, where the series values at one time step are affected by other time steps separated by a specific interval. On the other hand, aperiodic frequency domain features exhibit more local dependencies. In this case, values at one point in time may be influenced by neighboring values in time, but a consistent and predictable relationship may not exist over longer time periods.

Existing works still overlooks in predicting long sequences with multivariate influenced by the frequency domain, as long-term forecasts that incorporate multiple variables generally exhibit more complex temporal patterns compared to short-term forecasts, and failure to address this issue may result in the unreliable identification of temporal dependencies. Recently, Recently, Transformer-based architectures have exhibited superior performance in modeling sequential data through the application of self-attention mechanisms [10] that enables multivariate input forecasting models to make long-term predictions [11].

In practice, multivariable inputs can be analyzed and predicted to study how the signal changes over time and in the frequency domain. In the time domain, the signal focuses on global dependence on the data and insight into spectral properties. In contrast, frequency domain analysis aims to represent the time series in terms of frequency magnitude components. Allows the identification of local dependencies in the signal and transient behavior provision [12]. However, most existing studies on deep learning models primarily focus on extracting and utilizing temporal domain features from multiple sources of feature data, although certain models, such as informer [13] and FEDMormer [14], Utilizing a time-frequency transformation technique, such as the Fourier transform, However, these approaches primarily focus on reducing the time complexity of Transformer models to improve the training efficiency of the model, rather than making full use of the rich features in the frequency domain. Although Informer and FEDFormer have demonstrated effectiveness in leveraging the additional frequency domain characteristics provided by Fourier or discrete wavelet transforms, further improvements can be achieved through more refined feature fusion strategies, only the extracted frequency domain features are used as a complement to the time domain representation, the integration of features from two distinct domains fails to capture a comprehensive set of signal characteristics. Moreover, it will weaken the influence of frequency domain features and bring noise to the model.

In response to these challenges, this paper introduces a novel approach: the WOA-WTConv-KANformer model.The method employs the wavelet transform within an efficient architecture specifically tailored for the analysis of multifrequency properties of non-stationary time series data. Different from the existing wavelet method based on Fourier transform, the Wtconv2d convolution model [15] is used to integrate the wavelet transform of multi-medium frequency feature learning with the Transformer architecture, and Kan model [16] is used to replace the traditional MLP model as the linear layer of the Transformer architecture. The whale optimization algorithm [17] is used to optimize the hyperparameters before training, providing a more flexible and robust framework for analyzing time series data.

Our contribution can be summarized as:

In this paper, we propose a long sequence prediction model based on the Transformer framework that uses the Wtconv2d convolution model to optimize the multivariate input by extracting different frequency features in multiple channels, so that the model makes full use of the inherent characteristics of multivariate in the frequency domain and time domain.

In this paper, we propose to replace the MLP model with the KAN model as the linear layer of the Transformer framework, which achieves a better training fitting effect while reducing the number of training parameters.

Comprehensive experiments are carried out on long sequence prediction tasks in multivariable input time series problems. The experimental results demonstrate that the predictive performance of the proposed model is markedly superior to that of the prevailing state-of-the-art models.

## 2. Background and related work

The evolution of deep learning has significantly influenced the field of time series modeling and analysis. Recurrent neural networks (RNNs), including Long Short-Term Memory (LSTM) networks [18], are designed to capture temporal dependencies by utilizing hidden layers. The Multilayer Perceptron (MLP) [19] has proven to be effective in timing analysis through its ability to process sequential projections on a point-wise.For time series prediction, various deep sequence models are based on the classical sequence-to-sequence (seq2seq) framework for predicting multivariate inputs. DeepAR [20] integrates the concept of autoregression with RNNs to model the probability distribution of the predicted sequence. In addition to RNN models, convolutional neural networks (CNNS) are also used for multivariate input prediction. Such as, graph WaveNet [21] and TCN [22] use dilated causal convolutions to expand the field of view of the model and make the model only focus on historical information, thus obtaining a wider range of periods. In practice, the learning patterns and dependencies of these verified structures are based on the assumption that the consensus statistics feature may conflict with the non-stationary nature of time series data. On the other hand, most real-world time-dependent data exhibits non-stationary dynamics over time, thereby violating the stationarity assumption.

In recently,Transformer-based for multivariate input prediction models have received increasing attention with two strands of research have been conducted along this line. Log-Trans [23] and Autoformer, focus on replacing the original attention mechanism with a sparse attention mechanism, Both the time and space complexity of the algorithm are O(N^2^). On the other strand, such as Informer and Pyraformer [24], focuses on introducing various resolution representations of the original sequence by utilizing convolution operations or Fourier transforms, thereby capturing the time-domain characteristics of the sequence at different scales, thereby improving the attention mechanism at the level of the decomposition structure and thereby reducing the computational complexity. These decompositions are carried out in the time domain with the objective of reducing the sequence length, thereby enhancing computational efficiency.However, most of these decomposition methods are implemented in the time domain with the aim of reducing sequence length, thereby enhancing computational efficiency; hence, the information in the frequency domain, as a means to supplement or reduce the computational complexity, is not fully utilized.

In order to solve the above problems, frequency domain prediction methods have also been widely explored.The frequency domain provides a unique perspective on time series data by revealing the underlying periodicity and frequency components. In the field, multiple forecasting methods have recently emerged that are specifically designed to exploit these features and effectively capture the global dependencies inherent in time series data. Specifically, Autoformer substitutes the self-attention mechanism with an autocorrelation mechanism based on the principal period detected using the Fast Fourier Transform (FFT). TimesNet converts 1D time series into 2D space based on the principal period detected by FFT from topk spectral magnitude.The frequency improved model uses Legendre polynomial projection technology to approximate the historical information, and uses frequency enhancement method to extract frequency feature extraction. FreDo [25] proposed a simple yet powerful model called AverageTile that converts tiledseries to the frequency domain and uses DFT for more accurate extraction of features.In addition to another analysis of signal time-frequency characteristics of the technology: wavelet transform (WT) technology, is also widely used in the research of time series analysis [26]. The wavelet transform decomposes the signal into time and frequency domains. Therefore, it can capture the local periodic characteristics of time series more effectively. Specifically, for a time series signal x = [x0,..., xT −1], the transformation is defined as follows.


$$WT(s,\tau )=\int x_{t}\cdot \frac{\text{1}}{\sqrt{s}}\cdot \psi^{*}(\frac{t-\tau }{s})dt\;(0\leq t<T)$$ (1)

Where s represents the scaling factor, τ behalf of the translation factor, Ψ(t) is the chosen mother wavelet function, and * is the complex conjugate operation. The relation between Ψ(t) and Ψ(t)*is as follows.


$$\psi_{s,\tau }^{*}(t)=\frac{\text{1}}{\sqrt{s}}\psi (\frac{t-\tau }{s})$$ (2)

## 3. Method of woa-wtconv-kanformer model

### 3.1 Problem defintion

Given the historical data $x=[x_{1},...,x_{T}]\in R^{T\times D}$ in a multivariate time series,where T denotes the number of time steps in the historical data and D represents the number of variables. the objective of the time series prediction task is to forecast the values $y=[x_{T+1},...,x_{T+P}]\in R^{P\times D}$ for P future time steps.

### 3.2 Framework

**Fig 2:**
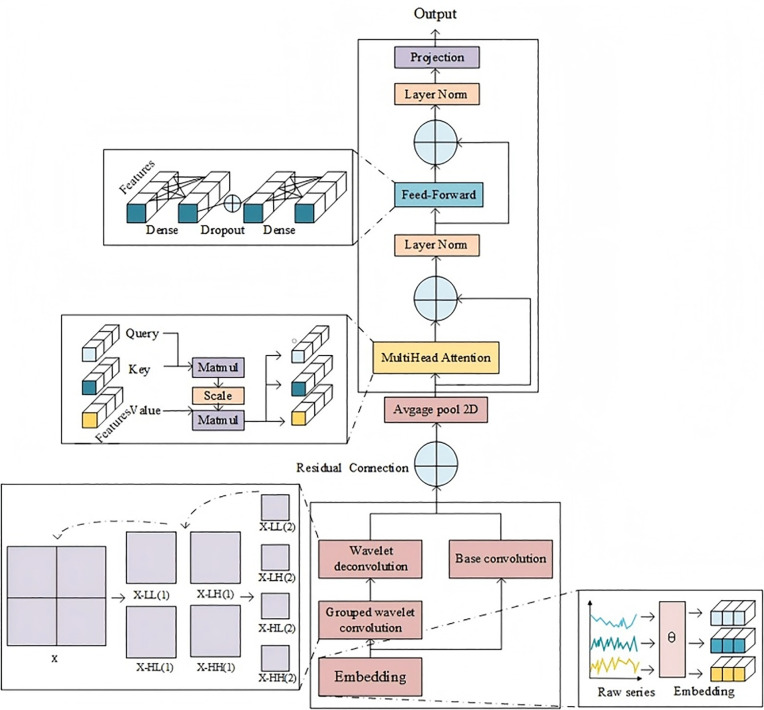
Overall structure of woa-wtconv-kanformer.

The WOA-WTConv-KANformer model uses the Transformer encoder framework, which is enhanced by WTConvBlocks before embedding data and input data into the encoder layer [27]. First normalizes the input matrix *x*_*in*_ to produce the *x*_*norm*_ matrix,then normalized data is transformed into the feature space *x*_*enc*_ ∈ *R*^*T×D*^. By normalizing along the time dimension, the data distribution is normalized to mean 0 and variance 1, so as to alleviate the non-stationarity of multi-feature time series data through normalization within the time window.After data normalization, the data is passed through a wavelet Convolutional block (WTConv), which converts the 1D time series into a 2D spatial representation. These blocks utilize efficient convolutional networks to divide the input time series data into multiple different frequency channels, thus effectively capturing local and global periodic features.Then, these feature matrices extracted by different signal features are input into the encoder layer of the Transformer framework for processing. The KAN model is employed to replace the traditional multilayer perceptron (MLP), based on the Kolmogorov-Arnold representation theorem, the multivariate continuous function can be expressed as the combination of univariate continuous function and addition operation [28], so as to avoid the need for exponential growth of neurons in traditional MLP. Reduce the number of model parameters.Then In order to obtain appropriate hyperparameters before training, the whale optimization algorithm is used to adjust the parameters of the model before the formal training.The model framework is presented in the Fig 2 below.

### 3.3 Whale parameter optimization algorithm

Models based on the Transformer framework involve the tuning of numerous hyperparameters, and inappropriate configurations can not only degrade model performance but also significantly extend training duration. Consequently, it is essential to determine optimal settings for critical hyperparameters prior to initiating the training process. To address this challenge, the Whale Optimization Algorithm can be employed to effectively optimize those parameters that exert a substantial influence on model performance [29].

The Whale Optimization Algorithm (WOA) is a bio-inspired computational technique that simulates the foraging behavior of humpback whales. The algorithm incorporates three primary mechanisms: encircling prey, spiral movement during hunting, and a random search strategy for exploring the solution space. Within the framework, the current best solution is treated as the target prey, and each search agent updates its position iteratively by applying these mechanisms. Model parameters are adjusted throughout successive iterations to converge toward the global optimal solution [30].

Encircling Hunting: In the initial phase, the randomly initialized parameters correspond to a locally optimal solution within the current model, referred to as the best search operator. Subsequently, other search operators iteratively adjust their positions toward that of the best search operator and update their respective parameters accordingly. Within the predefined n-dimensional search space, the global optimal solution remains unknown. The WOA treats the current best candidate solution as the target prey, as mathematically represented in Equations (3) and (4).


$$D=\left|C\cdot X^{*}(t)-X(t)\right|$$ (3)

$$X(t+1)=X^{*}(t)-A\cdot D$$ (4)

In equations (3) and (4), D denotes the distance vector between the search operator and the target object. The variable t represents the iteration number, while A and C denote random coefficient vectors used to compute the distance between the current operator and the target parameters. Specifically, A functions as a linearly decreasing vector that governs the encircling behavior during hunting and prey search processes. X*(t) signifies the optimal position vector, whereas X(t) represents the current position vector of the individual whale. The computational formulations for A and C are defined as follows [31]:


A=2a·r−a (5)

C=2·r (6)

Hunting behavior: During the hunting process, individual operators perform a local search in the vicinity of the current tentative optimal solution. By updating their positions through a spiral movement mechanism, they simulate the algorithm’s search for potential optimal solutions, thus achieving local optimization. The corresponding mathematical model is presented in Equations (7) and (8).


$$D=\left|X^{*}(t)-X(t)\right|$$ (7)

$$\begin{array}{c} X(t+1)=\{\begin{array}{c} {}*20cX^{*}(t)-A\cdot D,P<0.5\\ D\cdot e^{bl}\cdot \cos\theta +X^{*}(t),p>0.5 \end{array} \end{array}$$ (8)

D denotes the distance vector between the current optimal individual and the current individual within the operator group. The constant b represents the spiral coefficient, which governs the convergence trajectory of the operator. Here, l ∈ [−1, 1] and θ ∈ [−2π, 2π]. Furthermore, p (where p ∈ [0, 1]) acts as a conditional parameter that determines the position update strategy during the predation phase. Based on the random parameter P, the update mechanism is selected: if P < 0.5, the encircling hunting strategy is adopted; otherwise, the spiral-based position update mechanism is employed [32].

Search prey: To achieve convergence, this algorithm uses parameter A to control the stages of the operator population and implements the global search strategy through random search. When |A| > 1, the algorithm indicates that parameter information cannot be obtained. Instead, the current position of the operator group is the local optimal position, thereby ensuring that this process can conform to the global optimal solution. The formula for the search for prey process is as follows:


$$D=\left|C\cdot X_{rand}(t)-X(t)\right|$$ (9)

$$X(t+1)=X_{rand}(t)-A\cdot D$$ (10)

D denotes the distance between the current searching individual and the random individual, and Xrand(t) denotes the position of the current random individual [33].

**Fig 3:**
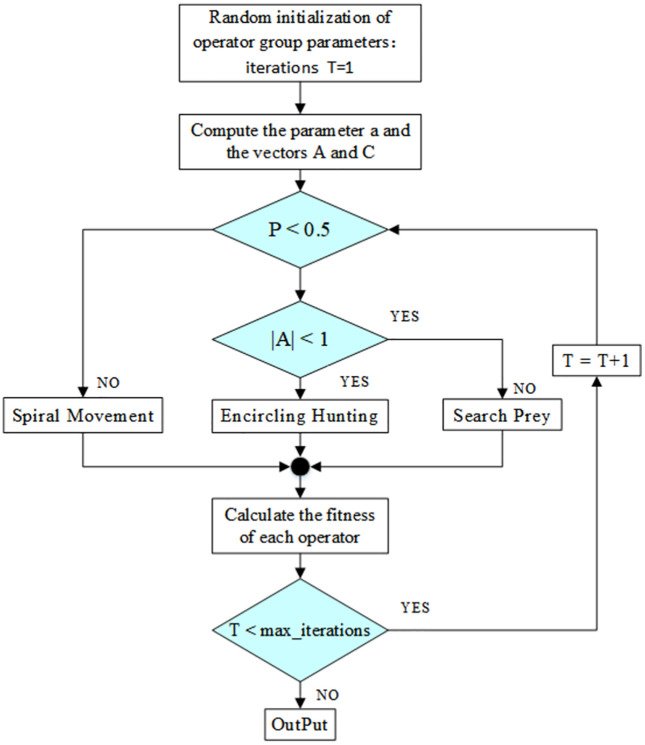
Flowchart of whale optimization algorithm.

Overall, the WOA algorithm first randomly initializes a set. During each iteration, the operator group randomly selects a parameter position of a certain operator or the relatively optimal solution obtained so far to update the parameter position.Reduce parameter A from 2 to 0 with each iteration, thereby gradually converging towards the tentative optimal solution. When |A| > 1, select the random search prey mode; when |A| < 1, update the parameter positions of the current local optimal solution update operator group.Depending on the value of p, the WOA algorithm can switch between spiral movement and Encircling Hunting. Finally, the WOA algorithm is terminated after reaching the number of iterations. The flowchart of the WOA algorithm is shown as Fig 3.

The execution process of parameter selection using the WOA algorithm is as follows: First, the algorithm randomly initializes a population of candidate solutions. The fitness of each solution is evaluated based on the specific loss function of the WTConv-KANformer model under the current set of parameters, and the solution associated with the minimum MSE loss is identified as the optimal solution. Subsequently,calculate the parameter a and the coefficient vectors A and C.

Subsequently, the task execution of the operator group within the WOA module is governed by the parameter |A| and the random parameter P. Specifically, when P < 0.5 and |A| < 1, the Encircling Hunting operation is initiated, during which the positions of the operators are updated to converge toward the current relatively optimal solution.When P < 0.5 and |A| ≥ 1, the Spiral Movement operation is executed, the parameters of the operator group are updated, and the spiral movement of whales around the prey is simulated using the spiral equation.When P ≥ 0.5, the Search Prey operation is carried out. A solution randomly selected from the operator group is updated to increase the diversity of the parameter selection results and avoid getting stuck in a local optimal solution.

Then fitness value of each individual is evaluated and compared against the previously stored optimal fitness value. If a superior solution is identified, it replaces the current optimal solution. Subsequently, an assessment is conducted to determine whether the maximum number of iterations has been reached. If the termination criterion is satisfied,the calculation concludes; otherwise, the process proceeds to the next iteration.The pseudo-code of WOA is presented in the Table 1 below.

**Table 1:** The Algorithm of WOA.

Algorithm of WOA
while count <= iterations: # Compute the dynamic linear parameters A and b, which decay iteratively a_linear_component = 2 – count*(2/iterations); b_linear_component = −1 + count*(−1/iterations) # Update the leader, who may be succeeded by a new individual. leader = **update_leader**(position, leader) # Update the leader # Update the population positions based on leader positions position = **update_position**(position, leader, a_linear_component, b_linear_component,**fitness_function**) fitness = **fitness_function**(position) # Calculate the population fitness # Update the leader again to ensure it reflects the current best candidate. leader = **update_leader**(position, leader) count += 1

### 3.4 WTConv module

In time series prediction models, the WTConv module conducts a tree structured divide and conquer multi scale analysis of the input features via wavelet transform, effectively expanding the receptive field of the convolution. Subsequently, it performs deconvolution operations to achieve parameter optimization and noise suppression, thereby providing higher – quality input features for the encoder layer.

The WTConv module performs cascaded wavelet decomposition on the input data. Each convolutional kernel focuses on features of different frequency bands to carry out the convolution operation. For a set of multi-feature data, a layer of Haar wavelet transform is conducted through deep convolution with convolutional kernels of [1]/√2 and [1, – 1]/√2. Subsequently, a two-dimensional Haar wavelet transform is applied for sampling operation with a sampling factor of 2. Specifically, the following four sets of filters are used for deep convolution.


$$f_{LL}=\frac{\text{1}}{\text{2}}\left[\begin{array}{cc} {}*20c1 & 1\\ 1 & 1 \end{array}\right],\begin{array}{c} {}*20cf_{LH}=\frac{\text{1}}{\text{2}} \end{array}\left[\begin{array}{cc} {}*20c1 & -1\\ 1 & -1 \end{array}\right],\begin{array}{c} {}*20cf_{HL}=\frac{\text{1}}{\text{2}}\left[\begin{array}{cc} {}*20c1 & 1\\ -1 & -1 \end{array}\right], \end{array}\begin{array}{c} {}*20cf_{HH}=\frac{\text{1}}{\text{2}}\left[\begin{array}{cc} {}*20c1 & -1\\ -1 & 1 \end{array}\right] \end{array}$$ (11)

*f*_*LL*_ as low-pass filter, whereas *f*_*LH*_, *f*_*HL*_, and *f*_*HH*_ are high-pass filters. For each input channel, the output of the convolution operation as:


$$\;[X_{LL},X_{LH},X_{HL},X_{HH}]=Conv(\left[f_{LL},f_{LH},f_{HL},f_{HH}\right],X)$$ (12)

The output consists of four channels, each featuring a resolution of *X/2* in each spatial dimension. Specifically, *X*_*LL*_ represents the *X* low-frequency components, while *X*_*LH*_, *X*_*HL*_, *X*_*HH*_ correspond to the horizontal, vertical, and diagonal components of the high-frequency information respectively.Cascaded wavelet decomposition is achieved through the recursive decomposition of low-frequency components. The decomposition at each level is expressed as follows:


$${X_{LL}}^{\left(i\right)},\;{X_{LH}}^{\left(i\right)},\;{X_{HL}}^{\left(i\right)},\;{X_{HH}}^{\left(i\right)}\;=\;WT\left({X_{LL}}^{\left(i-1\right)}\right)$$ (13)

Among them, *X*^*(0)*^_*LL*_ = *X*, and i represents the current decomposition level. The wavelet decomposition mechanism enhances the frequency resolution for lower frequencies while reducing the spatial resolution.

**Fig 4:**
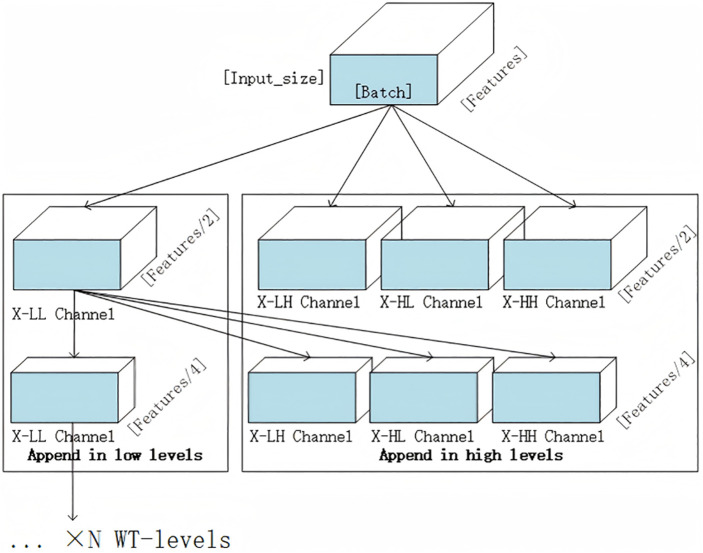
Diagram illustrating the grouped convolution within the wtconv module.

If the number of levels of the wavelet convolution surpasses 1, wavelet convolution persists in being carried out on the low – frequency output. Consequently, the size of receptive field is directly associated with the number of levels in the wavelet transform. As the number of layers L increases, The size of feature matrix in the current layer’s wavelet convolution becomes 2^L^ times smaller compared to the original feature matrix; via step-by-step recursion, the receptive field of the convolution kernel expands to the full-sized feature map. The schematic diagram of the grouped convolution is presented in Fig 4.

After filtering and downsampling the low-frequency and high-frequency components of the input via multi-channel wavelet convolution, the inverse wavelet transform (IWT) is employed to extract global features for the construction of the output. The process is delineated as follows.


Y=IWT(Conv(W,WT(X))) (14)

*X* represents the input matrix, while *W* denotes the weight tensor of a k × k convolution kernel with four times as many channels as the input matrix *X*. This operation decouples convolutions among frequency components, enabling smaller kernels to effectively operate over a broader region of the original input, thereby increasing the receptive field relative to the input size.The formula is presented as follows:


$${X_{LL}}^{\left(i\right)}\;,{X_{H}}^{\left(i\right)}\;=\;WT\left(X_{LL}{{}^{(i-1}}^{)}\right)$$ (15)

$${Y_{LL}}^{\left(i\right)},{Y_{H}}^{\left(i\right)}\;=\;Conv(W^{\left(i\right)},\;(\;X_{LL(i)}\;,\;X_{H(i)}))$$ (16)

Among them, *X*^*(i)*^_*LL*_ denotes the low frequency feature of the i-th layer, whereas *X*^*(i)*^_*H*_ denotes the three high-frequency features of the i-th layer. Subsequently, to effectively integrate the outputs of different frequencies, the linear properties of the wavelet transform (WT) and its inverse transform (IWT) are employed. These properties are used to integrate various features extracted by multi-channel wavelet convolution and restore the multi-feature data to its original size prior to wavelet processing, enabling the subsequent feature data to be input into the encoder layer of the time series model for further processing.

Specifically, the WTConv module conducts a divide-and-conquer recursive wavelet decomposition on each layer of the feature matrix and subsequently reassembles them gradually. Initially, it applies the wavelet transform to the low-frequency components. Subsequently, it executes convolution operations on different frequency components separately. Finally, it accumulates the results on each layer through the inverse wavelet transform (IWT). In this manner, the WTConv layer can capture different frequency information of the input at various levels, thereby attaining more effective feature extraction. The formula of the inverse wavelet transform (IWT) is derived by transposing the convolution kernel in Equation 1 to form an orthogonal basis, as presented below:


$$\begin{array}{c} X=Conv-transposed(\left[f_{LL},f_{LH},f_{HL},f_{HH}\right],\\ \left[X_{LL},X_{LH},X_{HL},X_{HH}\right]) \end{array}$$ (17)

**Fig 5:**
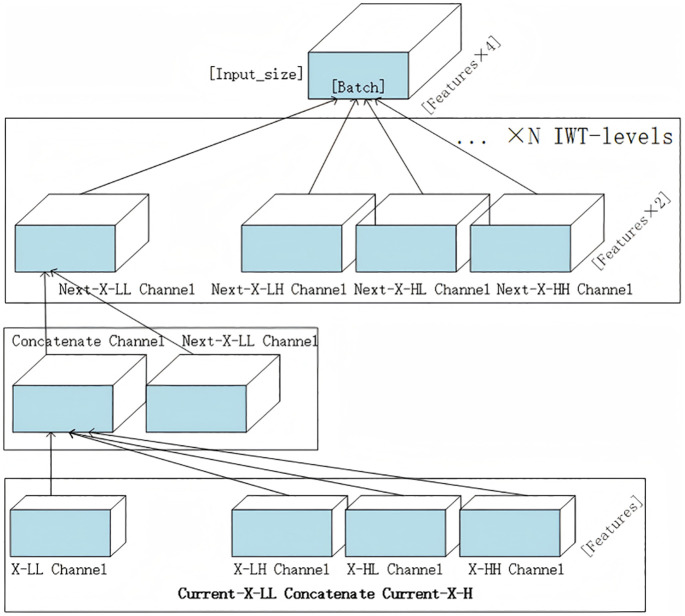
Illustration of wavelet deconvolution within the wtconv module.

An example diagram of wavelet recombination that performs gradually hierarchical accumulation of results through wavelet inverse operation is shown as Fig 5:

The pseudo-code of the algorithm for WTConv module, which conducts hierarchical wavelet convolution and subsequently reorganizes the features via wavelet deconvolution, in the Table 2:

**Table 2:** The Algorithm of WTConv model.

Algorithm of WTConv
**Input:** X # The input of original feature matrix XLL (0) = X **for** i = 1,..., ℓ **do #** Grouped WT-convolution of WTConv XLL (i), XLH (i), XHL (i), XHH (i) = Conv ([f_LL_,f_LH_,f_HL_,f_HH_], XLL (i-1)) **for** i = ℓ,..., 1 **do #** The IWT-inverse convolution reorganization operation of in WTConv XLL (i-1)= Conv-transposed ([f_LL_,f_LH_,f_HL_,f_HH_],[X_LL_(i),X_LH_(i),X_HL_(i),X_HH_(i)]) X = XLL (0) + X

### 3.5 KAN-based transformer for time series prediction

The Multilayer Feedforward Perceptron (MLP) is one of the most widely adopted and practically effective neural network architectures. However, despite its extensive application, the MLP model presents certain limitations, including a high demand for non-embedded parameters within the Transformer framework and reduced interpretability compared to attention-based layers [34]. In this paper, the MLP module is replaced with the KAN module, aiming to enhance linear fitting performance by integrating more interpretable components into the network and enabling the approximation of intermediate functions.

**Fig 6:**
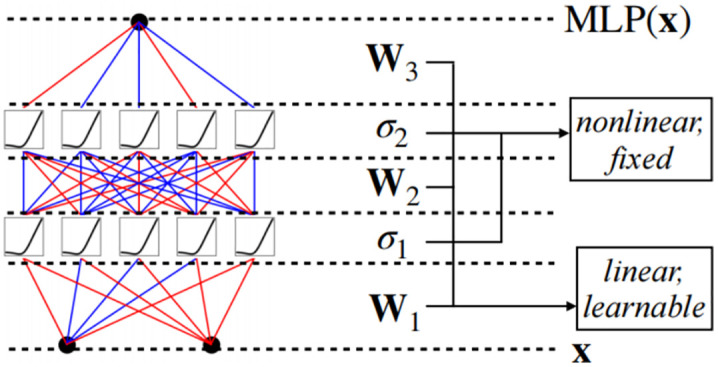
Linear layer of MLP network.

The traditional linear layer in a MLP network consists of multiple stacked fully connected layers. In each layer, the input data x undergoes a linear transformation defined by a weight matrix W and a bias vector b, resulting in z = Wx + b. The output z of linear transformation is passed through a nonlinear activation function σ,such as ReLU or Tanh, to introduce nonlinearity, yielding the activated output a = σ(z). The activated result a is used as the input of the next layer and passed layer by layer until the final output layer.The diagram of the MLP network is shown in Fig 6.

Consider a MLP network with n input units and m output units, where fully connected layers exist between layer l and layer l + 1.The equation can be expressed in matrix form as: x^(L+1)^ = W_(L+1,L)_x^(L)^ + b^(L+1)^. where W_(L+1,L)_ denotes the weight matrix connecting layer L to layer L + 1, x(L) represents the input vector to layer L, x^(L+1)^ denotes the output vector from layer L + 1, and b^(L+1)^ signifies the bias vector associated with layer L + 1. The weight matrix W_(L+1,L)_ is expanded as follows:


$$f\left(\text{x}\right)=\sigma ({\text{W}}_{\text{L}}\sigma ({\text{W}}_{\text{L}-1}\cdot \cdot \cdot \sigma \left({\text{W}}_{2}\sigma \left({\text{W}}_{1}\text{x}+{\text{b}}^{1}\right)+{\text{b}}^{2}\right)\cdot \cdot \cdot +{\text{b}}^{\left(\text{L}-1\right)})+{\text{b}}^{\text{L}})$$ (18)

In contrast to traditional linear layers based on MLP networks, KAN architectures are grounded in the Kolmogorov-Arnold representation theorem. In KANs, each weight is replaced by a learnable univariate function, and no fixed nonlinear activation function is applied at any node. Instead, nodes perform summation operations to aggregate these learnable functions, and the inter-layer transformations are modeled as adaptive spline functions rather than static activation functions, thereby enhancing the accuracy of function approximation. Moreover, since the intermediate layers in KANs consist of differentiable, non-fixed functions, gradient computation during backpropagation becomes smoother, which mitigates the issue of gradient vanishing commonly associated with fixed activation functions. This characteristic contributes to improved training stability, particularly in deeper network configurations.

In the KAN network, there are L linear layers, and x^L^ is an n-dimensional vector. By transposing this vector, x^L^ is embedded as a row in a matrix X with n columns: x^(L)^ ∈ R^n^ → (x^(L)^)^T^ ∈ R^1×n^, each row of X^L^ is the transpose of the vector (x^(L)^)^T^. subsequently define the operator *To* acting on the matrix Ψ_(L+1,L)_(X_L_). This operator computes the sum of the elements in each row of the matrix and outputs the resulting vector as *To*(Ψ_(L+1,L)_(X_L_)). Indeed, Ψ_(L+1,L)_ acts on the input vector x^(L)^, aggregates the results, and generates an output element defined as:X_(L+1)_ = Ψ_(L+1,L)_(X_L_). Therefore, when X_0_ is designated as the sole input vector, the output of the entire network after L layers can be expressed by the following formula.


$$f_{KAN}\left(X_{0}\right)=x^{\left(L\right)}=To\left(\Psi_{\left(L,L-1\right)}\left(X_{L-1}\right)\right)$$ (19)

**Fig 7:**
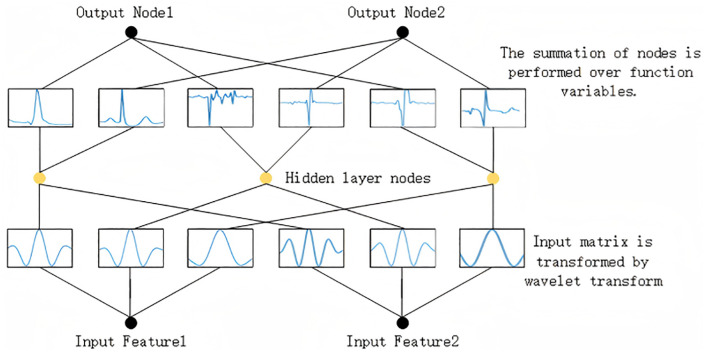
Linear layers integrated with wav-kan.

In this paper, the module employs a variant of KAN, referred to as Wave-KAN, capable of accommodating an arbitrary number of layers. The architecture of Wave-KAN is structurally analogous to that of MLP, with the key distinction being the replacement of traditional weights with wavelet functions [35]. Specifically, neurons in Wave-KAN aggregate inputs through learnable wavelet basis functions. Fig 7 depicts the configuration of Wave-KAN with two input features, three hidden nodes, and two output nodes.

After the input feature matrix is processed by the wavelet transform within the WTConv module, it is effectively decomposed into distinct frequency components.The components are subsequently fed into the KAN module, where scaling and translation of the wavelet basis functions are adaptively adjusted to capture high-frequency local details when the number of matrix features is large,or to focus on global patterns for trend identification when the feature count is small. By leveraging a set of scaled and translated basis functions, the module facilitates the accurate generation of both approximation and detail coefficients, enabling effective multi-resolution analysis.

Let Ψ ∈ L^2^(R) denote the mother wavelet and ø ∈ L^2^(R) represent the scaling function. The signal g(t)∈L^2^(R) can be decomposed into approximation coefficients a_*j*_(k) and detail coefficients d_j_(k)across j resolution levels as follows.


$$\begin{array}{c} {}*20ca_{j}(k)=\sum g(n)\varphi_{j,k}(n)\\ d_{j}(k)=\sum {\mathrm{\backslash nolimits}}_{n}^{n}g(n)\psi_{j,k}(n) \end{array}$$ (20)

g(n) denotes the discrete signal, ø_j,k_(n) represents the scaling function at scale j and location k, Ψ_j,k_(n) corresponds to the wavelet function at scale j and location k, a_j_(n) signifies the approximation coefficient, and d_j_(n) indicates the detail coefficient.

The original signal can be accurately reconstructed from its wavelet coefficients through the application of the inverse wavelet transform, ensuring that no information is lost during the transformation process. This reconstruction procedure is mathematically defined by the following formula:


$$g(n)=\sum_{k}a_{j}(k)\varphi_{j,k}(n)+\sum_{j=1}^{J}\sum_{K}d_{j}(k)\psi_{j,k}(n)$$ (21)

Within the framework of the KAN model, signals are represented as a superposition of wavelet functions across multiple scales and positions. Wavelet transformation enables the extraction of finer details by leveraging previously estimated coefficients. Furthermore, the inherent hierarchical structure of multi-resolution analysis ensures the reliability of these initial computations and facilitates parameter reuse.

## 4. Experiments

In this chapter, the experiment employed five extensively validated real – world datasets, namely the ECL, ETT (comprising four subsets), traffic, weather, and exchange rate datasets. The Mean Absolute Error (MAE) and Mean Squared Error (MSE) evaluation metrics were applied to compare the predictive capabilities of several mainstream models under different time series lengths. Meanwhile, the computation complexity of models and the modules they utilized were investigated. Not only has the proposed framework been verified to be effective in the time series prediction task, but also the controllability of the model in terms of performance overhead has been demonstrated. The evaluation metrics for MAE and MSE are as follows:


$$MAE=\frac{\text{1}}{n}\sum {\mathrm{\backslash nolimits}}_{i=1}^{n}|y_{i}-{\hat{y}}_{i}|$$ (22)

MAE represents the average of the absolute values of the errors, where n denotes the number of samples, and yi represents the true value, while ${\hat{y}}_{i}$ represents the predicted value.


$$MSE=\frac{\text{1}}{n}\sum {\mathrm{\backslash nolimits}}_{i=1}^{n}(y_{i}-{\hat{y}}_{i}{)}^{2}$$ (23)

MSE is calculated as the average of the sum of squared errors. Here, n represents the number of samples, yi denotes the true value, and ${\hat{y}}_{i}$ signifies the predicted value.

### 4.1 Performance comparison

In this subsection, to evaluate the prediction performance and Operational Costs of the proposed model in comparison with other advanced time series forecasting models, seven widely recognized prediction models are selected as benchmarks. These include: (1) Transformer based methods: Transformer, ITransformer, Informer, Kanformer, and PatchTST; (2) Dilated convolution based methods: TimesNet and TCN.

The prediction result indicators are presented in Table 3, and the indicator data with the best performance are highlighted in blue. Lower values of MSE and MAE signify more accurate time series prediction results of the model. In comparison with other predictors, the WOA - WTConv - KANformer model demonstrates excellent performance in predicting time series of moderate length, ranging from 96 to 336 data points. When compared to the Itransformer and KANformer models, which also exhibit good performance, the experimental data indicate that the time series prediction model integrated with wavelet convolution can incorporate the local changes in the frequency domain within the entire series, thereby enhancing its capability to capture and predict short-term frequency dynamics.

**Table 3:** Multivariate prediction results, model prediction length is S ∈ {96,192,336,720}, and fixed lookback length T = 96.

Models		Ours		Kanformer		Itransformer		TimesNet		PatchTST		Informer		Transformer		TCN	
		MSE	MAE	MSE	MAE	MSE	MAE	MSE	MAE	MSE	MAE	MSE	MAE	MSE	MAE	MSE	MAE
ETTh1	96	0.381	0.403	**0.374**	**0.397**	0.386	0.405	0.384	0.402	0.414	0.419	0.865	0.713	0.878	0.740	0.869	0.725
	192	**0.416**	0.457	0.419	**0.429**	0.441	0.436	0.436	0.429	0.460	0.445	1.008	0.792	1.037	0.824	1.023	0.814
	336	**0.458**	0.461	0.461	**0.450**	0.487	0.458	0.491	0.469	0.501	0.466	1.107	0.809	1.238	0.932	1.128	0.857
	720	0.481	**0.459**	**0.474**	0.467	0.503	0.491	0.521	0.500	0.500	0.488	1.181	0.865	1.135	0.852	1.175	0.858
ETTh2	96	0.305	0.351	0.301	0.353	**0.297**	**0.349**	0.340	0.374	0.302	0.348	3.755	1.525	2.116	1.197	2.876	1.457
	192	**0.375**	0.413	0.379	0.405	0.380	**0.400**	0.402	0.414	0.388	0.400	5.602	1.931	4.315	1.635	3.235	1.678
	336	**0.423**	**0.427**	0.432	0.446	0.428	0.432	0.452	0.452	0.426	0.433	4.721	1.835	1.124	1.604	1.136	1.542
	720	0.436	0.453	0.446	0.463	**0.427**	**0.445**	0.462	0.468	0.431	0.446	3.647	1.625	3.188	1.540	3.558	1.547
ETTm1	96	**0.317**	**0.354**	0.320	0.358	0.334	0.368	0.338	0.375	0.329	0.367	0.672	0.571	0.600	0.546	0.621	0.523
	192	**0.369**	**0.379**	0.364	0.383	0.377	0.391	0.374	0.387	0.367	0.385	0.795	0.669	0.837	0.700	0.784	0.687
	336	**0.397**	**0.401**	0.395	0.405	0.426	0.420	0.410	0.411	0.399	0.410	1.212	0.871	1.124	0.832	1.158	0.821
	720	0.498	0.463	**0.457**	**0.440**	0.491	0.459	0.478	0.450	0.454	0.439	1.666	0.823	1.153	0.820	1.135	0.821
ETTm2	96	**0.171**	**0.256**	0.176	0.261	0.180	0.264	0.187	0.267	0.175	0.259	0.365	0.453	0.768	0.642	0.405	0.523
	192	**0.227**	**0.298**	0.240	0.302	0.250	0.309	0.249	0.309	0.241	0.302	0.533	0.563	0.989	0.757	0.624	0.637
	336	**0.287**	**0.358**	0.299	0.342	0.311	0.348	0.321	0.351	0.305	0.343	1.363	0.887	1.334	0.872	1.328	0.865
	720	0.427	0.413	**0.397**	**0.401**	0.412	0.407	0.408	0.403	0.402	0.400	3.625	1.451	3.048	1.328	2.972	1.317
traffic	96	**0.387**	0.273	0.511	0.324	0.395	**0.268**	0.593	0.321	0.462	0.295	0.719	0.391	0.684	0.384	0.697	0.386
	192	**0.409**	**0.268**	0.529	0.330	0.417	0.276	0.617	0.336	0.466	0.296	0.696	0.379	0.685	0.390	0.678	0.381
	336	**0.427**	**0.278**	0.545	0.334	0.433	0.283	0.629	0.336	0.482	0.304	0.777	0.420	0.734	0.408	0.679	0.386
	720	0.482	**0.298**	0.580	0.351	**0.467**	0.302	0.640	0.350	0.514	0.322	0.864	0.472	0.717	0.396	0.735	0.428
weather	96	0.176	0.217	0.164	**0.210**	0.174	0.214	**0.172**	0.220	0.177	0.218	0.300	0.384	0.458	0.490	0.378	0.396
	192	0.215	**0.249**	**0.210**	0.251	0.221	0.254	0.219	0.261	0.225	0.259	0.598	0.544	0.658	0.589	0.569	0.635
	336	**0.261**	0.297	0.265	**0.290**	0.272	0.296	0.280	0.306	0.278	0.297	0.578	0.523	0.797	0.652	0.498	0.579
	720	0.353	0.345	**0.343**	**0.342**	0.358	0.349	0.365	0.359	0.354	0.348	1.059	0.741	0.869	0.675	0.658	0.859
exchange	96	**0.083**	0.213	0.097	0.215	0.086	**0.206**	0.107	0.234	0.088	0.205	0.847	0.752	0.968	0.812	0.876	0.795
	192	**0.175**	**0.289**	0.179	0.301	0.177	0.299	0.226	0.344	0.176	0.299	1.204	0.895	1.040	0.851	1.146	0.876
	336	0.342	0.408	0.347	**0.405**	**0.331**	0.417	0.367	0.448	0.301	0.397	1.672	1.036	1.659	1.081	1.658	1.157
	720	0.839	0.715	**0.834**	0.732	0.847	**0.691**	0.964	0.746	0.901	0.714	2.478	1.310	1.941	1.127	1.875	1.153
ECL	96	**0.143**	**0.238**	0.166	0.256	0.148	0.240	0.168	0.272	0.181	0.270	0.274	0.368	0.258	0.357	0.266	0.369
	192	0.172	0.263	0.187	0.274	**0.162**	**0.253**	0.184	0.289	0.188	0.274	0.296	0.386	0.266	0.368	0.275	0.378
	336	**0.173**	**0.259**	0.204	0.290	0.178	0.269	0.198	0.300	0.204	0.293	0.300	0.394	0.280	0.380	0.291	0.386
	720	0.238	0.321	0.247	0.323	0.225	**0.317**	**0.220**	0.320	0.246	0.324	0.373	0.439	0.283	0.376	0.297	0.385

**Fig 8:**
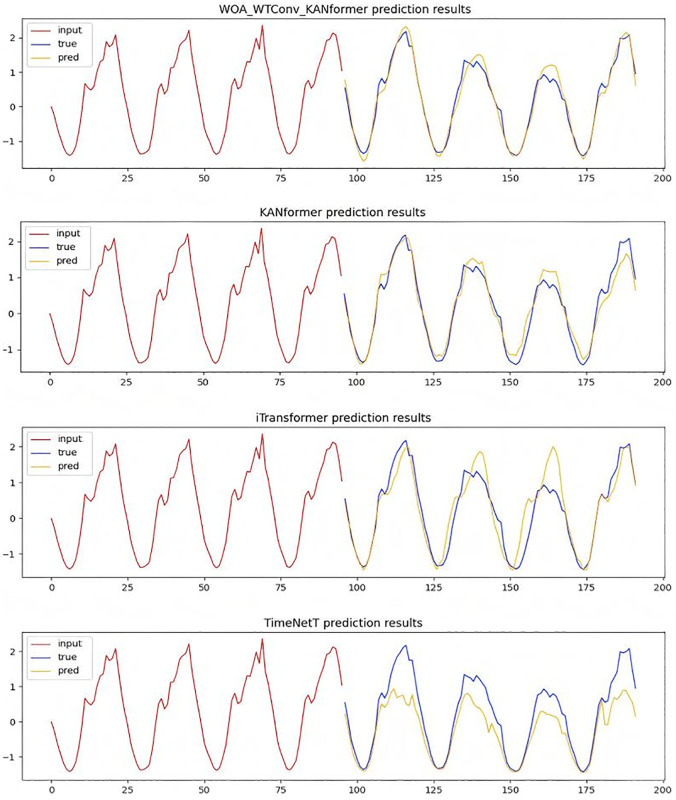
Time series prediction plot with a forecast horizon of 96 time steps.

**Temporal Image Analysis and comparison:** In order to verify the rationality of the WOA-WTConv-KANformer model in capturing local frequency domain information, detailed sample graphs were generated by utilizing a traffic dataset with 96 time steps. By comparing it with high-performance models based on the Transformer framework such as KANformer and Itransformer as well as the TimesNet model based on the dilated convolution framework, it has been proven that the WOA-WTConv-KANformer and KANformer model can effectively capture local frequency-domain information at medium and long time steps. The results are presented in Fig 8.

**Fig 9:**
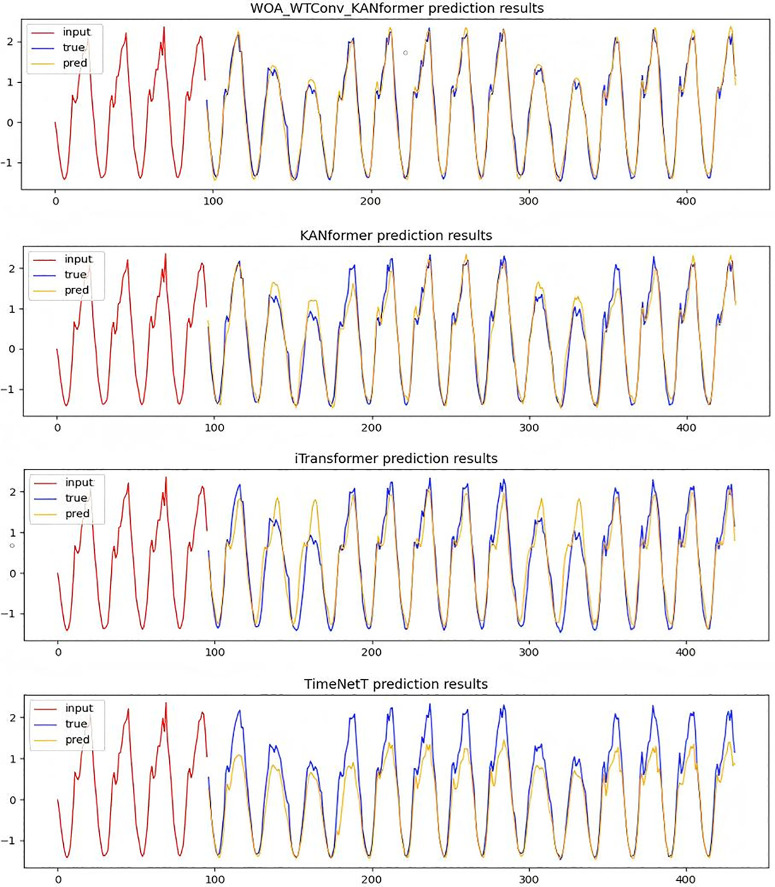
Time series prediction plot with a forecast horizon of 336 time steps.

In the Fig 8, the red portion represents the input real-time step data, the blue portion corresponds to the actual data to be predicted, and the yellow portion indicates the data predicted by the model. The greater the degree of overlap or proximity between the blue and yellow lines, the stronger the model’s ability to extract local frequency-domain features becomes. The predicted data from the WOA-WTConv-KANformer model align most closely with the actual data to be predicted, demonstrating superior performance in processing local information compared to other models.At the same time, the experiment employs a prediction task with a time step length of 336 to further validate the temporal accuracy and the capability of extracting local frequency-domain information of the prediction model under a longer time step.As depicted in Fig 9, the efficacy of the WOA-WTConv-KANformer model in extracting and predicting local frequency-domain information, as well as forecasting long-term time series trends, is validated.

Model efficiency: In order to comprehensively assess the performance of the model, taking the traffic dataset with a backtracking length of 336 time steps as an example, a comparison was made regarding the training speed, memory usage, the total number of parameters (Params) during model operation, and the number of floating point operations per second (Flops) of the model. The results are presented in Table 4.

**Table 4:** Comparison of model efficiency.

Models	Flops	Params	Training Time	Training memory
Ours	0.26 G	0.24 M	33 min	15.56 GB
Kanformer	0.22 G	0.21 M	28.5 min	13.52 GB
Itransformer	0.82 G	0.26 M	27 min	12.87 GB
Transformer	9.87 G	0.41 M	26.5 min	12.23 GB
TCN	0.81 G	0.19 M	35.5 min	17.28 GB
PatchTST	3.02 G	0.33 M	38 min	18.16 GB
Informer	42.35 G	1.89 M	31 min	12.68 GB
TimesNet	260.83 G	9.12 M	39 min	20.13 GB

Compared with the mainstream model based on the Transformer framework, the proposed model incorporates wavelet convolution to extract frequency domain characteristics prior to encoding the input. Although a slight increase the calculation of the parameters during the training period, the prediction accuracy of local frequency domain information is enhanced. Different from the TimesNet and PatchTST models, which are based on dilated convolution and convolution kernel merging, the proposed model does not demand to merge a large number of time series segments and stack multiple convolutional layers, thus reducing the amount of parameter calculation and training time.

### 4.2 Comparison of convolution modules

To verify the rationality of the convolution module selected by the model, the most suitable convolution module for processing the frequency domain and time domain feature matrices is identified. This subsection conducts experiments on the operation results and operation efficiency of the convolution module by replacing various convolution components.

**Model efficiency:** In order to comprehensively assess the performance of the model, taking the traffic dataset with a backtracking length of 336 time steps as an example, a comparison was made regarding the training speed, memory usage, the total number of parameters (Params) during model operation, and the number of floating point operations per second (Flops) of the model. The results are presented in Table 5.

**Table 5:** Performance comparison of each module.

Models	Flops	Params	Training Time	Training Memory
WTConv-Itransformer	0.29 G	0.15 M	29 min	14.32 GB
FFTConv-Itransformer	0.27 G	0.13 M	32.5 min	13.73 GB
TCN-Itransformer	0.61 G	0.18 M	37 min	19.26 GB

**Analysis of Convolution Modules:**To conduct a comprehensive evaluation of the performance of each convolutional module, this subsection presents an analysis of the computational complexity of each module, as depicted in Table 6. Simultaneously, by taking the traffic, weather, and ECL datasets with backtracking lengths of 336 and 96 time steps as examples, the impact of different convolutional modules on the model results is demonstrated, as shown in Table 7.

**Table 6:** Algorithm analysis of each module.

Components	Core algorithm	Computation complexity
WTConv	Multifrequency domain hierarchical feature extraction	O(n^2^logn*k^2^)
FFTConv	Fourier transformation extracts time domain and frequency domain information	O(n^2^logn)
TCN	Utilize dilated convolutions to expand the convolutional field of view	O(Mn²k²)

**Table 7:** Multivariate prediction results, model prediction length is S ∈ {96,336}, and fixed lookback length T = 96.

Models		WTConv-Itransformer		FFTConv-Itransformer		TCN-Itransformer	
		MSE	MAE	MSE	MAE	MSE	MAE
traffic	96	0.391	0.286	0.397	0.295	0.432	0.316
	336	0.431	0.286	0.436	0.301	0.532	0.298
weather	96	0.179	0.216	0.183	0.235	0.181	0.241
	336	0.273	0.305	0.265	0.297	0.261	0.310
ECL	96	0.159	0.247	0.147	0.253	0.163	0.267
	336	0.176	0.261	0.183	0.267	0.195	0.271

The Fourier convolution represents an efficient computational approach founded on the convolution theorem. It employs the Fast Fourier Transform (FFT) to transform the input feature matrix into time domain and frequency domain features, with a computational complexity of O(n^2^logn). Subsequently, the parameters of the two feature matrices are multiplied element by element to obtain residuals, and the processed features are inversely transformed to revert to the time domain features. Given that the FFT operation necessitates the storage of intermediate frequency domain results, the space complexity is O(n^2^).

Wavelet convolution represents a combination of wavelet transformation and convolution operation. It conducts convolution operations within sub-bands of varying scales (frequencies). In contrast to Fourier convolution, which first transforms the entire signal into the frequency domain and then performs a global multiplication operation. The complexity of wavelet convolution is contingent upon the complexity of wavelet transformation and hierarchical convolution operations. Each layer’s decomposition and reconstruction entail convolution and sampling. For an input time series signal, the computational complexity of layer by layer wavelet transformation is O(n^2^logn), the complexity of grouped convolution is O(k^2^), where k × k denotes the size of the convolution kernel, and the total computational complexity is O(n^2^logn*k^2^). The space complexity of wavelet convolution primarily arises from the requirement for temporary storage space during the decomposition, convolution, and reconstruction processes. The space complexity is O(n^2^).

The algorithmic mechanism of TCN primarily relies on dilated convolution and residual connections. By introducing the dilation coefficient d, “holes” are inserted between the elements of the standard convolution kernel. Here, d is a hyperparameter. When d = 1, it represents a regular convolution; when d = 2, the convolution kernel samples one point at every other point on the input, and the size of its receptive field expands from k to k + (k – 1) * (d – 1). A typical TCN consists of multiple residual blocks stacked together, and each residual block employs dilated convolution. The input data passes through the initial convolution layer, then enters a series of multiple residual blocks, and finally yields the prediction result via one or more fully connected layers. Regarding the computational complexity of TCN, assuming that the TCN network has M layers and each layer conducts a convolution operation on its input, the time complexity of forward propagation for the entire network is O(M*n²*k²).Given that the expansion convolution operation requires the storage of intermediate convolution results, its space complexity is O(n²).

**Fig 10:**
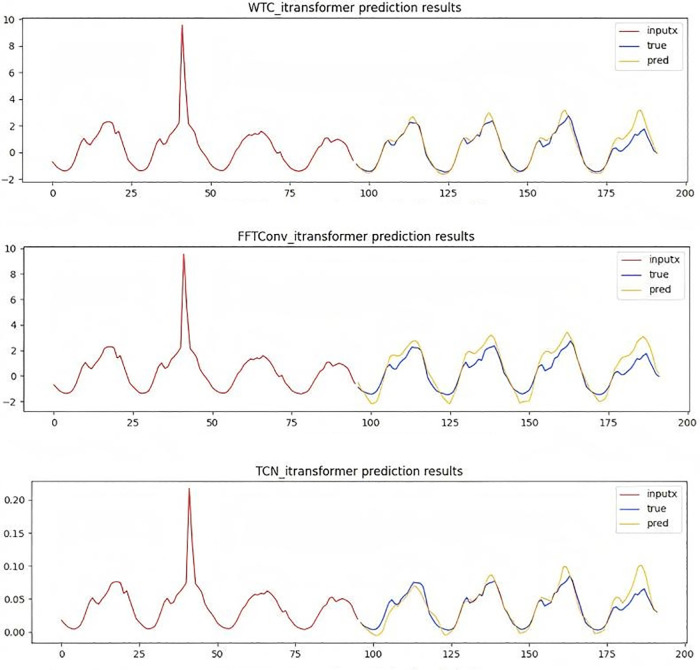
Time series prediction plot with a forecast horizon of 96 time steps.

As depicted in Fig 10, through a comparison of the model performance within the traffic dataset featuring a prediction step – size of 96, it visually illustrates the adaptability of the WTConv convolution module in dealing with temporal local frequency domain problems.

### 4.3 Ablation study

To validate the rationality of the components employed in this model, this subsection conducts experiments on component substitution and component removal. Through ablation experiments of various sub – modules, the calculation results and computational efficiency are verified.

**Model efficiency.**In order to conduct a comprehensive evaluation of the performance of each component, a traffic dataset with a backtracking length of 336 time steps was taken as an example. The training speed of the model, memory usage, the total number of parameters (Params) during the model’s operation, and the number of floating – point operations per second (Flops) were compared. The results are presented in Table 8.

**Table 8:** Performance comparison of each model.

Models	Flops	Params	Training time	Training memory
WTConv-KANformer	0.26 G	0.24 M	33 min	15.56 GB
WTConv-Itransformer	0.29 G	0.15 M	29 min	14.32 GB
KANformer	0.22 G	0.21 M	28.5 min	13.52 GB
Itransformer	0.82 G	0.26 M	27 min	12.87 GB

**Experimental indicators.** In order to reflect the performance outcomes of each module in the ablation experiment, this subsection utilizes the traffic, ECL, and weather datasets. The backtracking step size is set at 96, and the prediction step sizes are set at 96 and 336 respectively, and the results are presented as Table 9.

**Table 9:** Multivariate prediction results, model prediction length is S ∈ {96,336}, and fixed lookback length T = 96.

Models		WTConv-KANformer		WTConv-Itransformer		KANformer		Itransformer	
		MSE	MAE	MSE	MAE	MSE	MAE	MSE	MAE
traffic	96	0.387	0.273	0.391	0.286	0.511	0.324	0.395	0.268
	336	0.427	0.278	0.431	0.286	0.545	0.334	0.433	0.283
weather	96	0.176	0.217	0.179	0.216	0.164	0.210	0.174	0.214
	336	0.261	0.297	0.273	0.305	0.265	0.290	0.272	0.296
ECL	96	0.143	0.238	0.159	0.247	0.166	0.256	0.148	0.240
	336	0.173	0.259	0.176	0.261	0.204	0.290	0.178	0.269

**Fig 11:**
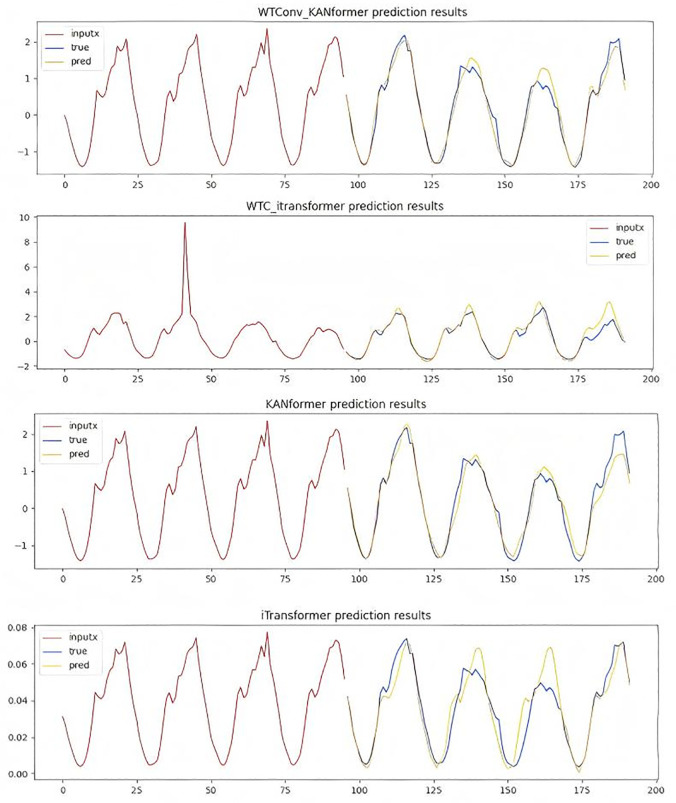
Time series prediction plot with a forecast horizon of 96 time steps.

**Fig 12:**
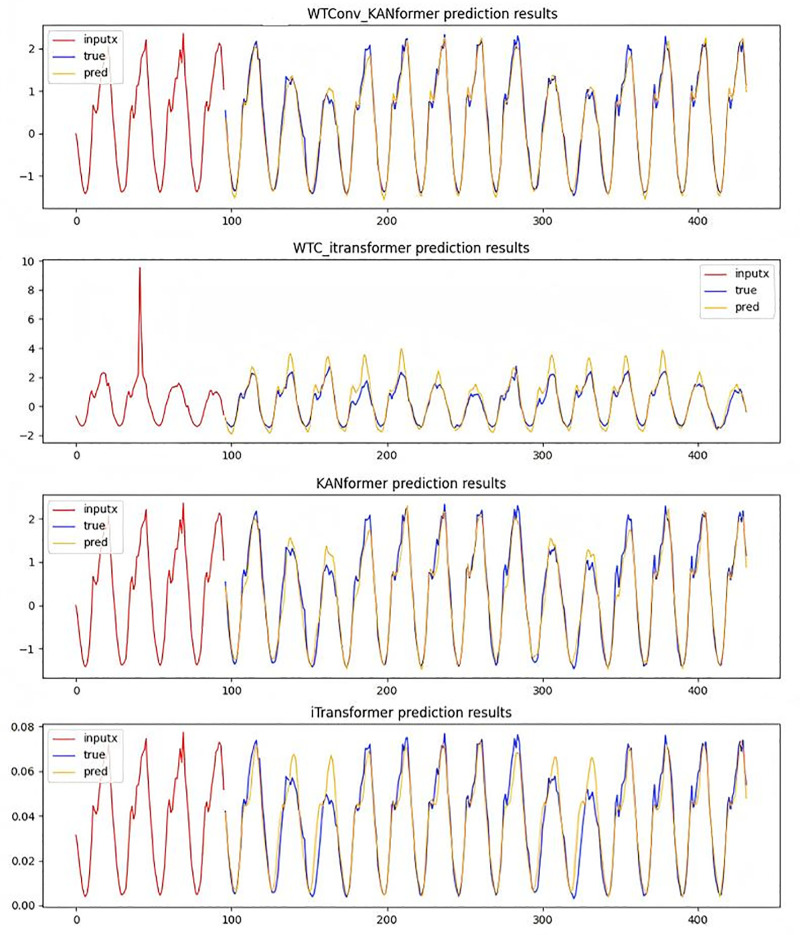
Time series prediction plot with a forecast horizon of 336 time steps.

To more intuitively demonstrate the effectiveness of the WTConv convolution module based on the Itransformer framework and the effectiveness of replacing the MLP module with the KAN module, the subsection presents the performance of various models in the traffic dataset at prediction steps of 96 and 336 in Fig 11 and Fig 12.

### 4.4 WOA module training optimization process

This subsection not only delineates the specific parameters influencing the WOA optimization model but also expounds on the recommended value ranges for each of the primary parameters in the WOA optimization algorithm. Additionally, it clarifies on which training parameters of the WTConv-KANformer model the WOA algorithm conducts parameter tuning iterations.

In the WOA algorithm, the following core parameters are defined: hunting_party (The number of operator groups). This parameter controls the diversity of the search process. A value that is too small may cause the algorithm to become trapped in local optima (specifically, if it is set below 10). To ensure an appropriate balance between global convergence and rapid iteration, it is recommended to adjust this parameter to a range of 20–30. For more complex problems, the value can be increased to 50.

Spiral Parameter: It is used for updating the linear attenuation parameters of Line A and Line B. The parameter governs the extent of rotation during the spiral search. The default range is from 0.5 to 1.5. An excessively large value might lead to oscillations, whereas an extremely small value will cause the search to proceed at a slow pace.

Iterations (iteration count): 2–3 times are insufficient for parameter convergence. It is recommended to have 5–10 iterations to balance training efficiency and convergence results; for models with excessive hyperparameters such as LLM, Diffusion and other technical stacks, the iteration count is suggested to be 10–15 times.

In this study, to select the appropriate hyperparameters for the model based on the efficiency of the balanced training, the default number of iterations for WOA is set to 5.Take the task of predicting with a step length of 96 using the weather dataset as example. By employing the MSE as the loss function, the final parameters of the WOA are selected according to the loss function value after the final iteration. The results are presented in Table 10.

**Table 10:** WOA parameter optimization loss function value.

WOA parameters	hunting_party: 10	hunting_party: 15	hunting_party: 20
Spiral Parameter: 0.5	0.381	0.379	0.368
Spiral Parameter: 1	0.376	0.374	0.352
Spiral Parameter: 1.5	0.362	0.365	0.357

The convergence times corresponding to different parameter selections are presented in Table 11.

**Table 11:** WOA parameter optimization time consumption.

WOA parameters	hunting_party: 10	hunting_party: 15	hunting_party: 20
Spiral Parameter: 0.5	7 min	8.5 min	8.5 min
Spiral Parameter: 1	7.5 min	8 min	10 min
Spiral Parameter: 1.5	6 min	7.5 min	9 min

By weighing the effectiveness of WOA parameter optimization and the time consumption, the final parameter selection for model optimization was hunting_party = 20 and Spiral Parameter = 1. The specific key parameters requiring fitting and adjustment are presented in Table 12.

**Table 12:** The list of parameters with their respective definitions.

Parameter designations	Explanation of parameters
e_layers	Number of Encoder Blocks
d_model	Model feature dimensionality
d_ff	Dimensions of the feedforward hidden layers
kernel_size	Size of the convolutional window
wt_levels	Number of Wavelet Convolution Layers
n_heads	Number of multi-head attention heads

**Fig 13:**
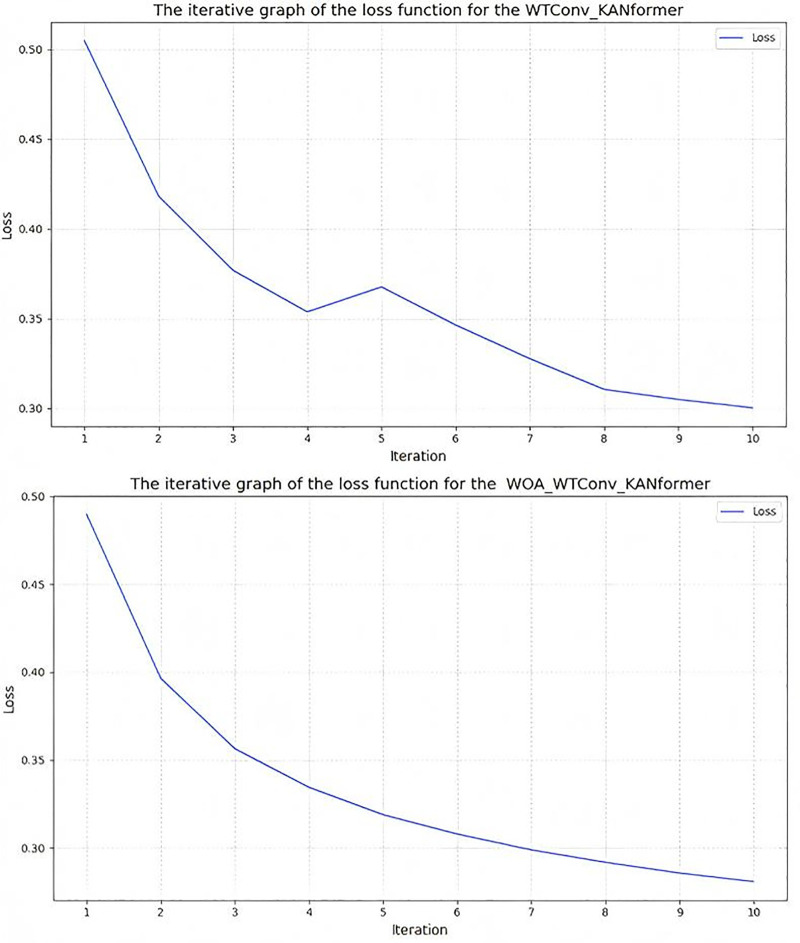
Iterative comparison of model loss function (mse) before and after woa optimization.

To confirm the enhancement contributed by the whale optimization algorithm module to the model’s training performance, the loss function of the model prior to applying the optimization algorithm was recorded. The results are illustrated in Fig 13.The parameter tuning process conducted during the WOA parameter tuning iteration is presented in Table 13.

**Table 13:** Parameter tuning iterative process.

Iteration	Loss(MSE)	e_layers	d_model	d_ff	kernel_size	wt_levels	n_heads
1	0.371	1	116	70	5	1	6
2	0.368	1	82	66	5	1	5
3	0.365	1	78	75	5	1	5
4	0.361	1	128	85	5	1	4
5	0.352	1	128	106	4	2	4

**Fig 14:**
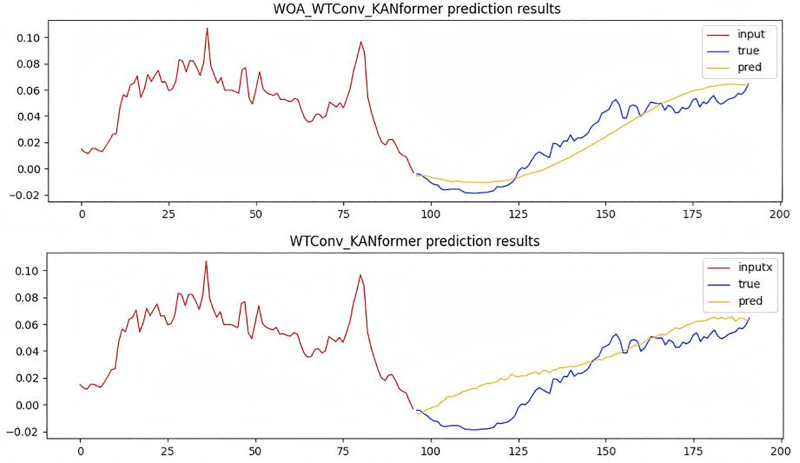
Prior and following the utilization of the WOA module.

To verify the effectiveness of the WOA parameter optimization module, as shown in Fig 14, the performance of WTConv-KANformer before and after using the WOA module was compared, which confirmed the feasibility of the WOA module in practical applications.

### 4.5 Generalization experiment on strong noise data

To verify the robustness of the model proposed in this paper in real-world scenarios, this section added Gaussian noise to the weather, traffic and ECL datasets, randomly deleted 20% of the data rows at each time step, and randomly selected 20% of individual data features from the remaining data rows for masking, and filling with column means. Through the prediction task on the data in the high-noise environment, the practicality of the model in extreme conditions is verified.

The formula for Gaussian noise is as follows: where Ps denotes the effective power of the signal, and Pn denotes the effective power of the noise. When the SNR value is higher, the noise level decreases. In the experiment of this subsection, the default value of SNR is set to 10.The model prediction indicators within a noisy environment are presented in Table 14.

**Table 14:** In a noisy environment multivariate prediction results, model prediction length is S ∈ {96,336}, and fixed lookback length T = 96.

Models		WTConv-KANformer		KANformer		PatchTST		TimesNet	
		MSE	MAE	MSE	MAE	MSE	MAE	MSE	MAE
traffic	96	0.721	0.687	1.031	0.783	0.924	0.736	1.136	0.813
	336	1.026	0.913	0.917	0.836	0.963	0.864	1.218	0.975
weather	96	0.326	0.357	0.358	0.371	0.309	0.326	0.372	0.392
	336	0.386	0.431	0.417	0.423	0.362	0.397	0.435	0.386
ECL	96	0.421	0.476	0.517	0.523	0.486	0.517	0.453	0.482
	336	0.657	0.625	0.716	0.654	0.683	0.637	0.735	0.674


$$\text{SNR}\;=\;\text{1}\text{0}\;{\text{log}}_{\text{1}\text{0}}{\text{P}}_{\text{s}}/{\text{P}}_{\text{n}}\;$$ (24)

**Fig 15:**
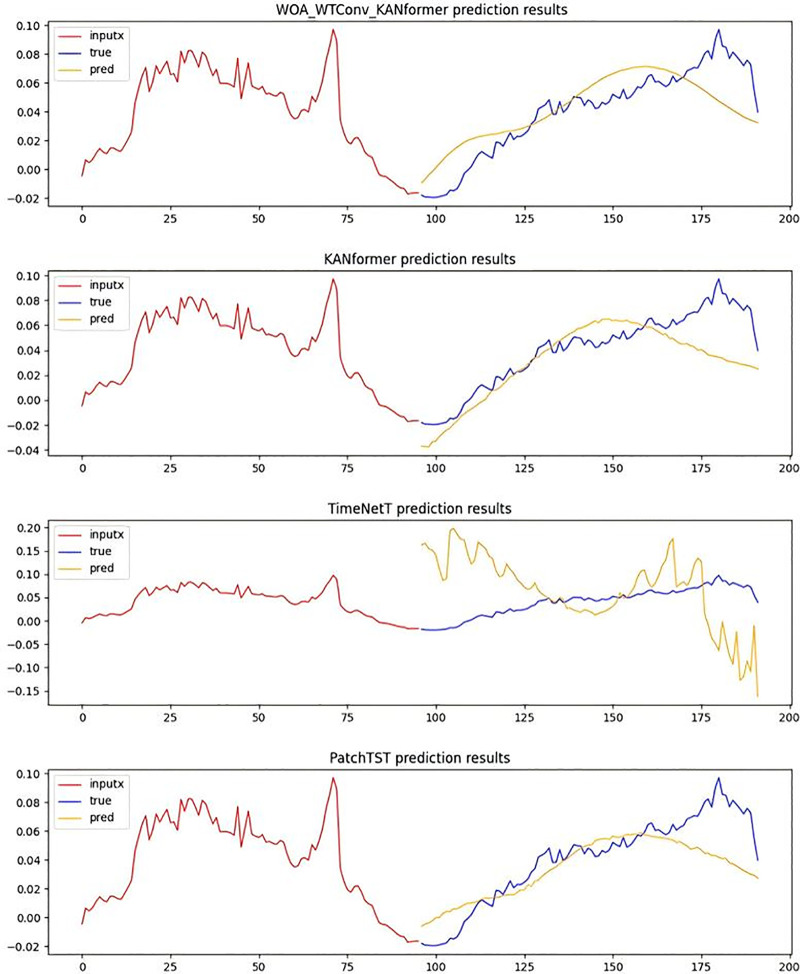
In a noisy environment multivariate prediction results, model prediction length is 96, and fixed lookback length T = 96.

**Fig 16:**
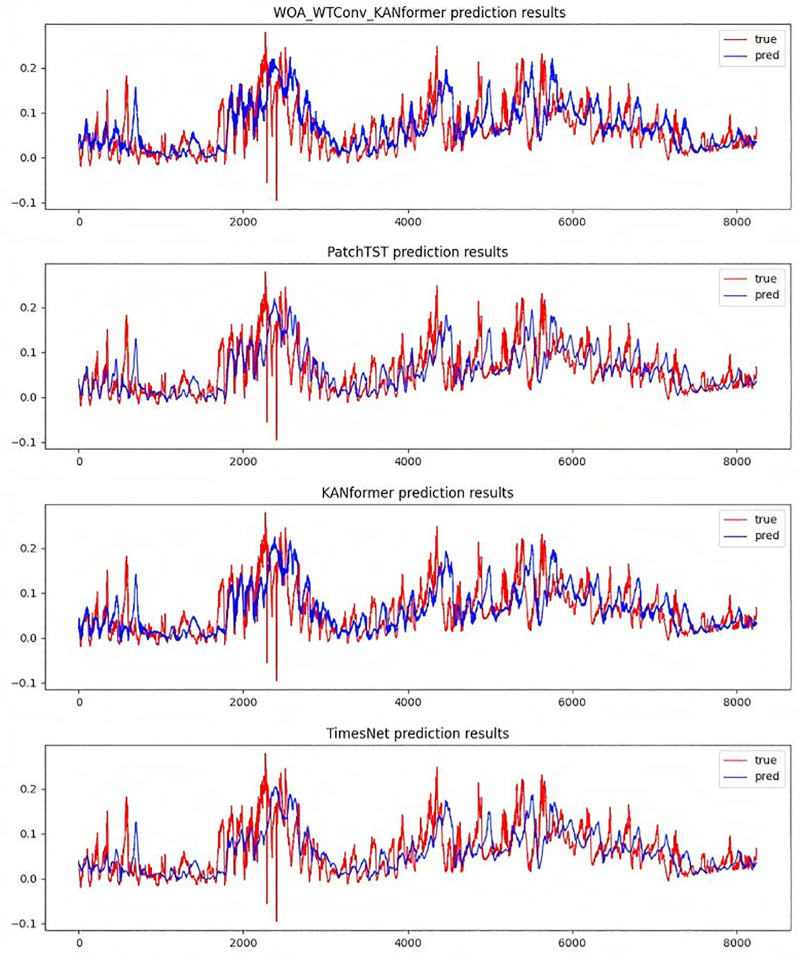
In a noisy environment multivariate prediction results,The global prediction graph with a prediction step size of 96.

To visually assess the performance of the WTConv - KANformer model in a high – noise environment, an example is provided using the weather dataset after noise processing. The results are presented via a prediction graph with a prediction step size of 96, as depicted in Fig 15.The global prediction results are shown in Fig 16.

Experiments have demonstrated that unprocessed datasets characterized by high noise and interference can exert a certain level of interference on the time – series prediction model. Nevertheless, the WTConv - KANformer model is capable of making relatively accurate predictions and trend reconstructions in both the frequency domain and the time domain.

## 5. Conclusion

In order to enhance the accuracy of local frequency domain information in time series prediction, this paper proposes WOA-WTConv-KANformer. The model integrates the strengths of time-domain prediction based on the Transformer framework and employs wavelet convolution to capture multi-frequency domain information prior to inputting data into the encoder layer. This enables the prediction model to balance the accuracy of both time-domain and frequency-domain predictions. Additionally, the KAN module is utilized as a linear layer to strengthen the model’s fitting capability to the data. Experimental results demonstrate that WOA-WTConv-KANformer exhibits advanced performance in time series prediction tasks and possesses a generalizable framework.
